# Surgical management of a patient with traumatic tension pneumorachis: A rare case report

**DOI:** 10.1016/j.ijscr.2019.12.035

**Published:** 2020-01-09

**Authors:** Abubaker AlAieb, Saji Mathradikkal, Atirek Goel, Gustav Strandvik, Mohammed Ellabib, Syed Nabir, Ahammed Mekkodathil, Hassan Al-Thani, Ayman El-Menyar

**Affiliations:** a Department of Surgery, Trauma Surgery, Hamad General Hospital, Doha, Qatar; b Department of Radiology, Hamad General Hospital, Doha, Qatar; c Clinical Research, Trauma & Vascular Surgery, Hamad General Hospital, Doha, Qatar; d Clinical Medicine, Weill Cornell Medical College, Doha, Qatar

**Keywords:** Pneumorachis, Trauma, Case report, Rectal injury, Neurologic deficit

## Abstract

**Introduction:**

Pneumorachis is a rare underdiagnosed phenomenon in which air enters the spinal canal mostly following injury to the respiratory system.

**Presentation of case:**

We presented a rare case of pneumorachis associated with neurological deficits following traumatic compressed air insufflation to the rectum. A 44-year-old, male, construction laborer presented to the accident and emergency department with a history of insufflation by compressed air through his rectum, as a prank by his workmates. Imaging studies revealed pneumoperitoneum and the exploratory laparotomy showed perforation of the sigmoid colon as well as a serosal tear of the transverse colon. Resection of the sigmoid by GI staplers was done. The patient remained unwell even after surgical management and massive transfusion and eventually succumbed to his injuries on the second day in the hospital.

**Conclusion:**

Our case suggests that pneumorachis should be considered one of the various causes for sublesional post-traumatic neurological deficits and mortality.

## Highlights

• This is a rare case of tension pneumorachis associated with neurological deficits following rectal insufflation.
• Perforation of the sigmoid colon and serosal tear of the transverse colon were diagnosed by exploratory laparotomy.
• Second laparotomy revealed diffused oozing from the left para-colic gutter, sigmoid colon and mesentery of the small bowel.
• Resection of the sigmoid by GI staplers was done.
• Patient succumbed to his injuries on the second day in the hospital.

## 1 Introduction

Pneumorachis is referred to a phenomenon where air enters into the spinal canal which is a rare event when compared to the pneumocephalus (intracranial air) [1]. This condition typically occurs following injury to the respiratory system or after traumatic brain injury especially with skull base and sinus fractures and very rarely from primary spinal sources, visceral injuries, and fulminant infections [1]. In most of the instances, it was not associated with any spinal cord symptoms and got resolved by dealing with the principal cause. However, pneumorachis may be an indication of spinal cord compression in trauma patients [2]. Symptomatic pneumorachis may be associated with various symptoms ranging from radicular pain to serious neurological deficits [3]. Advent of Computed Tomography (CT) contributed in increasing reported pneumorachis cases, however, it is difficult to detect in trauma settings and therefore remains as a diagnostic challenge [1,3,4]. Since pneumorachis is rare and occurs due to diverse etiologies, a definitive guideline for treatment is still lacking. We present a rare case of jet insufflation rectal injury resulting in symptomatic air within the spinal canal (Traumatic Tension Pneumorachis) which resulted in death of the patient. This case report has been reported in line with the SCARE criteria [5].

## 2 Case report

A 44-year-old, male, construction laborer, presented to the accident and emergency department (ED) with a history of insufflation by compressed air through his rectum, as a prank by his workmates during their works. Within thirty minutes of exposure to insufflation by compressed air, the victim started complaining of abdominal distension, diffused abdominal pain, difficulty in breathing and vomiting. The Emergency Medical Services (EMS) was called and reached the scene within 10 min. The patient was found supine, conscious with Glasgow Coma Score (GCS) 15/15 and normal vital signs. Significant abdominal distension was evident. He was noted by the EMS crew to have no movement in his lower limbs and his penis was semi-erect (priapism).

**Fig. 1:**
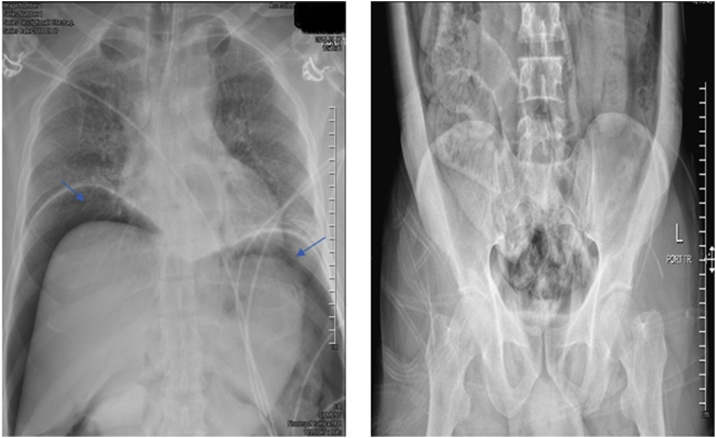
(A) Pneumoperitoneum (air under the diaphragm, blue arrow) (B) air extending to intra peritoneal and retroperitoneal region.

The patient was transferred to a level I trauma center, fully conscious and oriented with normal vital signs apart from mild tachypnea with 30–40 breaths/minute. During the assessment and resuscitation in the trauma unit, the patient was noted to have 0/5 power in the lower limbs, with loss of soft touch sensation to the level of the umbilicus. The priapism persisted. An extended focused assessment with sonography in trauma (e-FAST) was negative, and the chest X-ray revealed significant free air under the diaphragm (pneumoperitoneum) (Fig. 1).

**Fig. 2:**
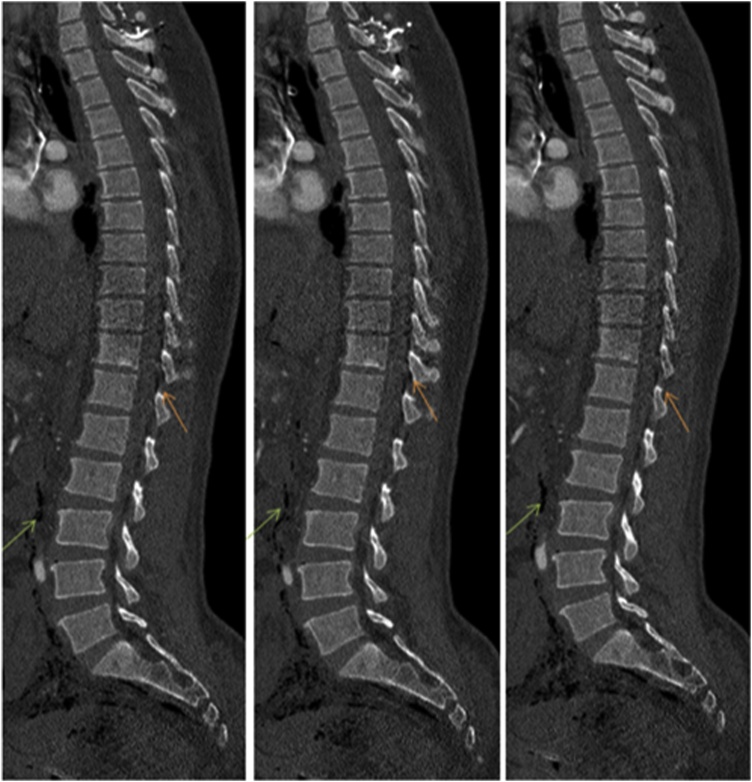
Sagittal sections of the dorsolumbar spine show free air in the spinal canal (orange arrow) and retroperitoneum (green arrow).

**Fig. 3:**
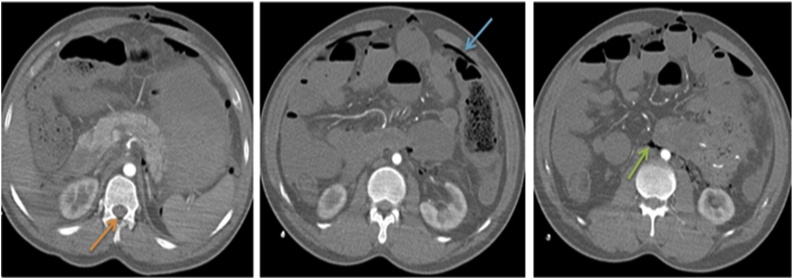
Axial images of the abdomen in soft tissue window shows free air in the spinal canal (orange arrow), intraperitoneum (blue arrow) and retroperitoneum (green arrow).

**Fig. 4:**
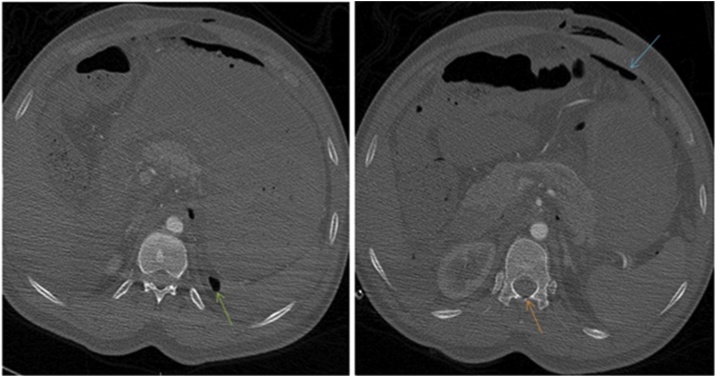
Axial images of the abdomen in bone window images show free air in the spinal canal (orange arrow) and retroperitoneum (green arrow).

The patient was taken immediately to the operating room (OR) for exploratory laparotomy. Operative findings included a perforation of the sigmoid colon as well as a serosal tear of the transverse colon. A segmental resection of the sigmoid colon with side-to-side anastomosis was performed, and the serosal tear was repaired. The skin was closed but fascia kept open. The patient was then transferred to the CT scanner to complete his imaging studies. The CT scan showed multiple pockets of air in the intraperitoneal, retroperitoneal and spinal canal spaces (Fig. 2, Fig. 3, Fig. 4).

The patient was transferred to the trauma intensive care unit (TICU) for continued resuscitation. He was hypotensive and the massive transfusion protocol (MTP) was activated to receive multiple blood and blood components. However, he remained hypotensive despite the aggressive fluid resuscitation and high dose inotropes. The primary surgery team decided to take him back to OR for re-exploration. The patient remained unstable and the CT showed a possible active bleeding in the pelvis. At the second laparotomy on the next day, he was found to have diffused oozing from the left para-colic gutter, sigmoid colon and mesentery of the small bowel. Resection of the sigmoid by GI staplers was done, the abdomen and pelvis were packed, and abdomen closed utilizing the ‘poor man’s technique’. The patient remained unwell in the TICU. Full resuscitative measures, including 5 shipments of MTP were continued. Unfortunately the patient succumbed to his injuries on the second day in the hospital.

## 3 Discussion

To the best of our knowledge tension pneumorachis following rectal insufflation trauma has not been reported before, in the English language.

Compressed air is used in almost every industry such as plants, ship yards and manufacturing industries. The risk of associated pneumatic injuries especially from its use while playing practical jokes is of major concern since it results in colorectal injuries which remain as a challenge for the surgeon. Spillage of the gut contents into the peritoneum following pneumatic injuries causes peritonitis, sepsis and may lead to death [6]. Ali et al. reported 10 cases of colorectal injures due to rectal insufflation while playing practical jokes at workplace and the patients were presented with signs and symptoms of peritonitis [6]. In those cases, X-rays and ultrasonography revealed dilated colon and gas under the diaphragm. None of them had respiratory distress. Explorative laparotomies were performed in all patients following the preoperative resuscitation. Perforation was sutured and proximal colostomy was performed in cases with multiple perforations. Uneventful recovery was reported in all cases [6]. We presented a rare case of pneumorachis, in which air enters the spinal canal, associated with neurological deficits following traumatic compressed air insufflation to the rectum. The patient was a victim of practical joke by his coworkers which ended up in the death of the patient in the hospital on the second day of admission.

Pneumorachis has also been described in the medical literature under various terms such as aerorachia or epidural emphysema [2,7]. It is most commonly encountered in the extradural, but may also extend within the subarachnoid space with disruption of the surrounding dura mater spinalis [2,4]. The most frequent causes of pneumorachis are either iatrogenic etiologies (peridural anesthesia, lumbar puncture or spinal surgery) or traumatic etiologies, the later are rare and include skull base fracture, spinal injuries, pneumomediastinum, pneumothorax, and insufflations injury [2,8]. Chaichana et al. conducted a review of literature and identified 50 cases of pneumorachis [1]. It was developed after injury to the respiratory system in the majority of cases (n = 28) followed by intracranial injury (n = 10). However, it was developed from a primary spinal source in 6 cases and from an intra-abdominal source in 5 cases while one patient developed pneumorachis spontaneously without any antecedent event [1]. Most of the cases were asymptomatic and neurological deficit was very uncommon but it was evident in our case.

Diagnosis modalities include CT scan and Magnetic Resonance Imaging (MRI) of the spine to detect the presence of low density intra-spinal air. MRI has high diagnostic efficacy which reveal contusion or any compression factor such as hematoma, hernia or bone fragment. However, clinical presentation is the corner stone of diagnosis [9]. Treatment includes non-operative and operative management [1]. Patient should also undergo serial imaging studies to follow up the decrease or increase of intra-spinal air. Most cases improve spontaneously with non-operative treatment including continuous high inspired oxygen therapy to achieve nitrogen washout. The non-operative management includes maintaining the patient position in the supine or Trendelenburg position, administration of prophylactic antibiotics in case of suspected contamination or open fracture, frequent neurological assessment and repeat CT scan if clinical deterioration occurs [1,2,10]. Operative treatment in symptomatic or deteriorated cases involves decompression of intra-spinal air [1,2,10]. The management should be decided on an individual basis, and through a multidisciplinary approach and should be aimed at identification of the cause, and prevent morbidity and mortality [10]. No mortality has been reported as a direct sequelae of pneumorachias per se, however, associated injuries may result in death as reported in our case.

## 4 Conclusion

Tension pneumorachis after rectal insufflation trauma has not been previously described. Our case suggests that pneumorachis should be considered as one of the various causes of sublesional post-traumatic neurological deficits and mortality.

## Funding

None.

## Ethical approval

This case report was granted approval from the medical research center at hamad medical corporation IRB#(MRC-04-19-304).

## Consent

Written informed consent was not obtained from the patient. The head of our medical team has taken responsibility that exhaustive attempts have been made to contact the family and that the paper has been sufficiently anonymised not to cause harm to the patient or their family. A copy of a signed document stating this is available for review by the Editor-in-Chief of this journal on request".

## Author contribution

Abubaker AlAieb MD, Saji Mathradikkal MD, Atirek Goel MD, Gustav Strandvik MD, Mohammed Ellabib MD, Syed Nabir MD, Ahmed Mekkodathil MD, Hassan Al-Thani MD, Ayman El-Menyar MD have substantial contribution in terms of study concept, data collection, surgical therapy for this patient, writing and approving the manuscript.

## Registration of research studies

Not applicable.

## Guarantor

Abubaker AlAieb.

## Provenance and peer review

Not commissioned, externally peer-reviewed.

## Declaration of Competing Interest

None.
